# Accelerated Intermittent Theta Burst Stimulation for Suicide Risk in Therapy-Resistant Depressed Patients: A Randomized, Sham-Controlled Trial

**DOI:** 10.3389/fnhum.2016.00480

**Published:** 2016-09-27

**Authors:** Stefanie Desmyter, Romain Duprat, Chris Baeken, Sara Van Autreve, Kurt Audenaert, Kees van Heeringen

**Affiliations:** ^1^Department of Psychiatry and Institute for Neuroscience, University Hospital GhentGhent, Belgium; ^2^Department of Psychiatry and Medical Psychology, Ghent UniversityGhent, Belgium; ^3^Unit for Suicide Research, Department of Psychiatry and Medical Psychology, Ghent UniversityGhent, Belgium

**Keywords:** repetitive transcranial magnetic stimulation, suicide, suicidal ideation, depression, therapy-resistant depression, theta burst stimulation

## Abstract

**Objectives:** We aimed to examine the effects and safety of accelerated intermittent Theta Burst Stimulation (iTBS) on suicide risk in a group of treatment-resistant unipolar depressed patients, using an extensive suicide assessment scale.

**Methods:** In 50 therapy-resistant, antidepressant-free depressed patients, an intensive protocol of accelerated iTBS was applied over the left dorsolateral prefrontal cortex (DLPFC) in a randomized, sham-controlled crossover design. Patients received 20 iTBS sessions over 4 days. Suicide risk was assessed using the Beck Scale of Suicide ideation (BSI).

**Results:** The iTBS protocol was safe and well tolerated. We observed a significant decrease of the BSI score over time, unrelated to active or sham stimulation and unrelated to depression-response. No worsening of suicidal ideation was observed. The effects of accelerated iTBS on mood and depression severity are reported in Duprat et al. (2016). The decrease in suicide risk lasted up to 1 month after baseline, even in depression non-responders.

**Conclusions:** This accelerated iTBS protocol was safe. The observed significant decrease in suicide risk was unrelated to active or sham stimulation and unrelated to depression response. Further sham-controlled research in suicidal depressed patients is necessary. (Clinicaltrials.gov identifier: NCT01832805).

## Introduction

Worldwide, over 800,000 people die of suicide every year and the number of non-fatal suicide attempts is estimated to be more than twenty times higher (WHO, 2014 report). Except for electroconvulsive therapy (ECT), few treatments for suicidal ideation and behavior (e.g., lithium, ketamine and clozapine) are available and these are only partially effective (Sher et al., 2010). Furthermore, the utility of ECT is limited due to safety concerns and adverse events. There thus is a great need for more convenient interventions with a rapid effect on suicide risk in order to prevent suicide.

Repetitive Transcranial Magnetic Stimulation (rTMS) has well-documented positive effects on depression without major side effects (Schutter, 2009). A review of the literature indicates that rTMS improves several preconditions for suicide, including mood, memory, attention, executive functioning and other neuropsychological dysfunctions, such as choosing immediate reward over larger, delayed rewards (Figner et al., 2010; Sher et al., 2010). Moreover, rTMS is thought to have molecular effects similar to those seen with ECT, which is also applied in suicidal patients, such as increased BDNF, increased monoamine turnover and normalization of the hypothalamic-pituitary-adrenal axis (George, 2010). Several researchers have reported an acute decrease of suicidal ideations following treatment with rTMS (O’Reardon et al., 2007; Holtzheimer et al., 2010; Hadley et al., 2011; Keshtkar et al., 2011; Wall et al., 2011; Desmyter et al., 2014; George et al., 2014). These results however were considered as preliminary because all but one were not sham-controlled while mostly very limited suicide assessment measures were used and sample sizes were relatively small. Thus, these preliminary results need to be substantiated by sham-controlled studies in larger groups of patients with more elaborate suicide assessment and longer follow-up. A systematic review and meta-analysis by Brunoni et al. (2009) indeed reported that placebo-response in depression is large in rTMS and antidepressant trials. In the only rTMS study that has used a sham-control, a significant decrease in suicide risk was observed but no significant difference between the TMS and the sham group (George et al., 2014).

Furthermore, previous rTMS research in depression has shown a dose-response relationship and more recently, studies have shown a trend towards administering more stimuli over a shorter period of time and at higher frequencies leading to accelerated protocols (Holtzheimer et al., 2010; Baeken et al., 2013; George et al., 2014). In addition, Theta Burst Stimulation (TBS) uses bursts of high frequency stimulation at repeated intervals and is thought to affect brain function more thoroughly when compared to “classic” rTMS (Huang et al., 2005; Di Lazzaro et al., 2008). Intermittent Theta Burst Stimulation (iTBS) induces a long-term potentiation (LTP)-like effect by increasing the postsynaptic concentration of calcium ions (Huang et al., 2009, 2011; Oberman et al., 2011). TBS has been applied to depression in several studies (Chistyakov et al., 2010; Li et al., 2014): it appeared to be safe and well tolerated and to have antidepressant properties.

For the current randomized, sham-controlled cross-over study, we aimed to examine the effect of accelerated iTBS on suicide risk in a group of treatment-resistant unipolar depressed patients, using an extensive suicide assessment scale. We hypothesized that this intensified treatment protocol would be safe in depressed patients with suicide ideation and would result in significant decreases in suicide risk in the active and not in the sham condition. To our knowledge this is the first study investigating accelerated iTBS as an intervention to reduce suicide risk.

### Subjects and Methods

This registered study was approved by the ethical committee of the Ghent University Hospital and all subjects gave written informed consent. It was part of a larger project investigating the effects of accelerated iTBS on depressive symptoms and suicide risk (clinicaltrials.gov identifier: NCT01832805). Part of this data set, including 12 patients, was also used in a conference article reporting preliminary results on the effects of accelerated iTBS on the SSI (Beck scale for suicidal ideation; Desmyter et al., 2014). The effects of accelerated iTBS on mood and depression severity are reported in Duprat et al. (2016).

Patients were screened using the structured Mini International Neuropsychiatric Interview (MINI; Sheehan et al., 1998). The 17-items Hamilton Depression Rating Scale (HDRS) was administered by an independent rater, and patients were required to have a minimum score of 14, which is defined as at least moderate depression (Hamilton, 1967). Patients were at least stage I therapy-resistant depressed, according to the Thase and Rush staging model, indicating the failure of at least one adequate trial of one major class of antidepressants (Thase and Rush, 1997). Depressed patients were diagnosed, screened and included several weeks before baseline because, when approved, antidepressant and antipsychotic medication and mood stabilizers were tapered off and stopped 2 weeks before the start and during the whole period of the iTBS treatment in order to evaluate the treatment in monotherapy.

**Figure 1:**
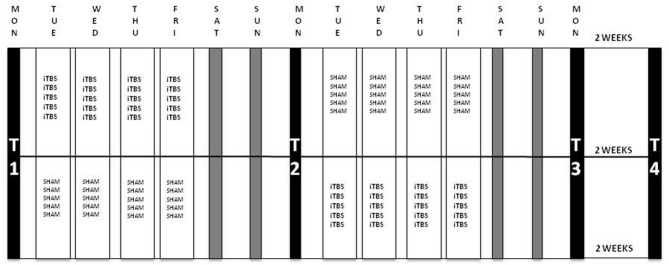
**Flowchart of the iTBS-sham-controlled cross-over protocol.** Patients were randomized to two groups: group A (*n* = 22) received iTBS during the first week and sham in the second week and group B (*n* = 24) was administered sham stimulation during the first and iTBS in the second week. Stimulation parameters: 110% resting MT (rMT), 1620 pulses per session in 54 bursts of three with a train duration of 2 s and an intertrain interval of 8 s; five sessions per day during 4 days per week. Evaluations were carried out at four time-points: at baseline (T1), after the first week of stimulation (T2), after the second week of stimulation (T3) and 2 weeks after the last stimulation, i.e., 1 month after baseline (T4). iTBS, intermittent Theta Burst Stimulation; MT, Motor Threshold.

Fifty therapy-resistant antidepressant-free patients were included (35 females), with a mean age of 41.90 years (SD = 11.77). Patients with the following conditions were excluded: psychotic symptoms, history of epileptic insult, cerebral surgery, having a pacemaker, having had ECT, alcohol dependence and patients who committed a suicide attempt within 6 months before the start of the study. Bipolar and psychotic depressed patients were not included. The 21-items Beck Scale for Suicide Ideation (BSI) was used to assess the intensity of the patients’ suicide risk. The BSI is a self-rating scale that measures the current intensity of the patients’ suicidal ideations, intentions and plans to commit suicide. Each item consists of three options graded according to suicidal intensity ranging from 0 to 2. The total score is yielded by the sum of the ratings for the first 19 items, ranging from 0 to 38. The BSI consists of five screening items. Three items assess the wish to live or the wish to die and two assess the desire to attempt suicide. If the subject reports any active or passive desire to commit suicide, then 14 additional items are administered. These consist of suicidal risk factors such as the duration and frequency of ideation, sense of control over making an attempt, number of deterrents, and amount of actual preparation for a contemplated attempt. Two additional items record incidence and frequency of previous suicide attempts (Beck et al., 1988; Beck and Steer, 1991). There were four time-points for evaluation at which depression severity was evaluated using the HDRS, assessed by a trained, but independent rater who was blind to the treatment condition (active or sham). The BSI was administered to evaluate suicide risk. These four time-points were: at baseline (T1), after the first week of stimulation (T2), after the second week of stimulation (T3) and 2 weeks after the last stimulation, i.e., 1 month after baseline (T4). Six months after baseline, patients were contacted to assess whether or not they had committed suicide. Patients were randomized to two groups: during the first week one group received the active stimulation and the other group started with the sham condition. They were switched to the other condition during the second week; see Figure 1 for a flowchart of the study design.

Intermittent TBS stimulation was applied using a Magstim Rapid^2^ Plus^1^ magnetic stimulator (Magstim Company Limited, Wales, UK) with a figure-of eight-shaped coil. The resting motor threshold (rMT) of each individual was determined on the right abductor pollicis brevis muscle before the first treatment session (Fitzgerald and Daskalakis, 2012). A stimulation intensity of 110% of the patient’s rMT was administered during treatment. We used the Brainsight neuronavigation system (Brainsight^TM^, Rogue Research, Inc.) to identify the site of stimulation (i.e., the center part of the midprefrontal gyrus [Brodmann 9/46]) based on a structural cerebral MRI of each individual in order to accurately target the left dorsolateral prefrontal cortex (DLPFC). A structural MRI of the brain was performed on a 3T Siemens^®^ Magnetom Trio MRI scanner (Siemens, Erlangen, Germany). All subjects underwent a T1-weighted MRI scan of the brain (3D-TFE, TR/TE = 2530/2.58; flip angle = 7°; FOV = 220 × 220 mm^2^; resolution = 0.9 × 0.9 × 0.9 mm^3^; number of slices = 176) with a 32 channel SENSE head coil. iTBS was delivered at five sessions per day during 4 days. One iTBS session consisted of 54 trains of 10 bursts of three stimuli. These stimuli were applied in a 50 Hz frequency: the bursts were repeated every 200 ms. This resulted in 2 s of stimulation alternated by 8 s rest periods and 1620 stimuli per session. With a total of 20 sessions, this yielded a sum of 32,400 stimuli per complete treatment. For the sham condition, a specially designed sham coil, looking completely identical as the active coil and making a similar noise but without delivering any active stimulation, was placed exactly on the same target region in the same position. Throughout the whole treatment (iTBS and sham), patients were blindfolded, wore earplugs and were kept unaware of the type of stimulation. Between two sessions, there was a pause of 15 min.

### Statistical Analysis

All data were analyzed using Statistical Package for the Social Sciences (SPSS; IBM SPSS Statistics for Windows, Version 23.0, IBM Corp., Armonk, NY, USA). The significance level was set at *p* < 0.05 for all analyses.

To examine the effect of the treatment protocol on suicide risk, mixed linear regression analyses were performed on the BSI scores with patient as random factors and Order (iTBS-sham or sham-iTBS) and Time as fixed factors. To further evaluate whether the main effect on suicidal ideation could not be attributed to a general improvement of depressive symptoms, we carried out mixed linear regression analyses on the BSI scores with factors Time and HDRS responder (on T4). Patients were considered HDRS-responders when they showed at least a 50% decrease of HDRS score. We applied a similar regression model replacing the dichotomous variable with the continuous changes in HDRS scores. To evaluate the differences in BSI score between the different groups at baseline, we applied independent-samples-*t*-tests. *Post hoc* paired *t*-tests were done on the BSI scores at the different time-points for all mentioned groups.

## Results

Three patients were considered drop-out from the study. One male patient erroneously received two times the active stimulation. One female patient spontaneously improved after antidepressant washout and was not stimulated. Another female patient was considered dropout from the study because of a suicide attempt (medication overdose) after the first week of sham iTBS. Data on one additional patient were not included in the analysis because the baseline BSI score was missing.

Of the 46 patients, 32 subjects reported suicidal ideations at baseline (T1), which was defined by a BSI score of larger than 1 (Beck et al., 1999). Consequently, all analyses were performed on these 32 suicidal subjects. The mean BSI score of the group that had suicidal thoughts (*n* = 32) was 13.31 (SD = 6.78). Eighteen patients received sham in the first week while 14 received active iTBS treatment during the first week. Both groups switched to the other condition for the second week. There was no significant difference in baseline BSI scores between the different groups (see Table 1).

**Table 1:** **Beck scale of suicide ideation (BSI) scores**.

	BSI							
	T1		T2		T3		T4	
	Mean	(95%CI)	Mean	(95%CI)	Mean	(95%CI)	Mean	(95%CI)
Total group	13.31	(10.52–16.13)	8.22	(5.08–11.61)	7.53	(4.57–10.50)	5.26	(2.47–8.24)
iTBS—sham	13.43	(9.21–17.64)	9.36	(4.46–14.26)	7.57	(3.12–12.02)	5.00	(0.90–9.62)
sham—iTBS	13.22	(9.51–16.94)	7.33	(3.02–11.65)	7.50	(3.57–11.43)	5.44	(1.67–9.22)

*Mean BSI scores and 95% CI on time-point T1, T2, T3 and T4 for the suicidal subgroup (n = 32)*.

No seizures, hypomanic or manic switches or other serious adverse events nor suicide attempts were observed during the treatment phase. Local discomforts at the stimulation site during treatment or headache during or after the session were mentioned, but these complaints disappeared spontaneously after a couple of hours or a single intake of paracetamol. There were no drop-outs caused by intolerance. None of the iTBS treated patients committed suicide up to 6 months after treatment. This was the last time-point of contact with the participants.

**Figure 2:**
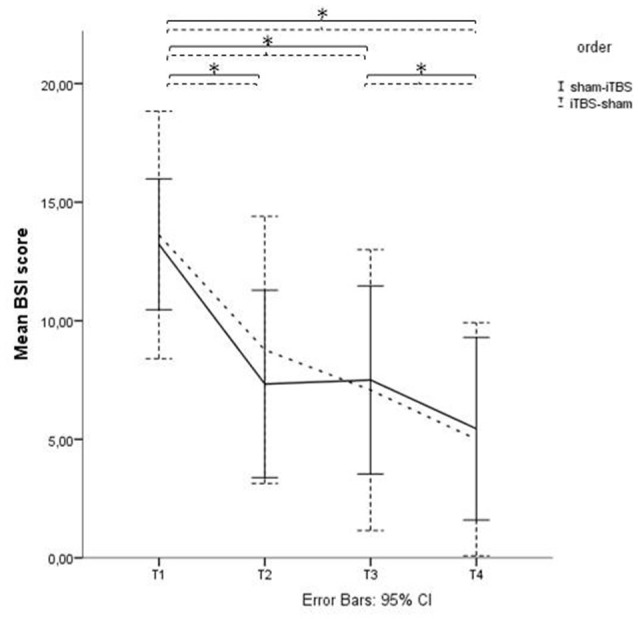
**Beck Scale of Suicide ideation (BSI) scores before and after active and sham treatment.** Graphical representation of the BSI mean scores with Time (baseline (T1), after 1 week of stimulation (T2), after finishing the treatment protocol (T3) and 2 weeks later (T4)) as within-subjects variable, and Order (sham > active vs. active > sham) as between-subjects factor. *Indicates significant difference.

Analyses showed a significant decrease of BSI scores over time (*p* < 0.01) between T1 and T2. There was no significant effect of the order of treatment (sham-iTBS vs. iTBS-sham; *p* = 0.66) and no significant interaction effect (*p* = 0.45; see Figure 2).

**Figure 3:**
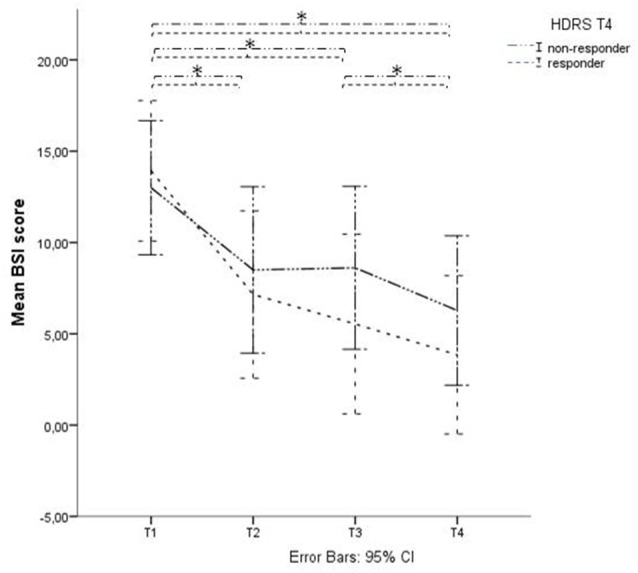
**BSI score in Hamilton Depression Rating Scale (HDRS)- responders and non-responders.** Graphical representation of the BSI mean scores with Time (baseline (T1), after 1 week of stimulation (T2), after finishing the treatment protocol (T3) and 2 weeks later (T4)) as within-subjects variable and HDRS responders on T4 as between-subjects factor. *Indicates significant difference.

At time-point 4 (T4), i.e., 1 month after baseline and 2 weeks after finalization of the treatment protocol, 18 of the 46 therapy-resistant depressed patients showed a depression-response, as defined by a decrease of at least 50% of their HDRS scores. This means that 39% of these therapy-resistant depressed patients responded to the stimulation protocol regarding their depressive symptoms (mean HDRS T1 = 21.50 (SD = 5.57); mean HDRS T4 = 12.83 (SD = 7.33)). Within the suicidal subgroup, 13 out of 42 patients showed a decrease of at least 50% of the HDRS scores at time-point T4, which equals 31%. When we divided this subgroup into HDRS-responders and HDRS-non-responders to evaluate the effect on suicide risk, the mixed linear regression model showed a significant decrease in BSI score in both groups, lasting up to T4 with a significant effect of Time (*p* < 0.01) but no significant effect of the factor “HDRS-response on T4” (*p* = 0.378) and no significant interaction effect (*p* = 0.19; see Figure 3). An independent-samples-*t*-test showed no significant difference in BSI score at baseline between the group of HDRS-responders and HDRS-non-responders (*t*_(30)_ = 0.42, *p* = 0.68). Applying a similar regression model replacing the dichotomous variable with the continuous changes in HDRS scores showed similar results: no significant interaction was found between time and HDRS change (*p* = 0.20).

*Post hoc* paired *t*-tests on the suicidal subgroup showed a significant decrease of BSI scores (*p* < 0.05) between T1 and T2, T1 and T3 and between T1 and T4, unrelated to active or sham stimulation and unrelated to depression response at T4 (defined as a 50% decrease of HDRS score).

In those patients (*n* = 14) with no suicide risk at baseline (BSI ≤ 1) we found no significant main effect of Time and no significant interaction effects indicating that there was no significant change in BSI scores in this non-suicidal subgroup.

## Discussion

This sham-controlled study shows a decrease of suicide risk following accelerated iTBS in treatment-resistant unipolar depressed patients, however, without statistically significant difference between the effect of active and the sham treatment. The antisuicidal effect lasted up to 1 month after baseline and appeared to be independent of the antidepressant effect. None of the iTBS treated patients committed suicide until 6 months after treatment. In line with other intensive rTMS treatment studies, our accelerated iTBS protocol was safe as only decreases in suicidal ideation were observed (Holtzheimer et al., 2010; Hadley et al., 2011; Baeken et al., 2013; George et al., 2014). Furthermore, in patients without suicidal ideation at baseline no significant increases in BSI scores were found. To our knowledge this is the first study investigating accelerated iTBS as an intervention to reduce suicide risk.

The lack of a significant difference between the active and the sham group after 1 week appears to be in line with the findings of George et al. (2014) who did not find a significant difference in effect between the active and sham group after 3 days of rTMS. Nevertheless, it is clear that the sham group also showed a significant decrease in suicide ideation after 1 week, indicating that placebo effects may influence the outcome in such iTBS studies.

The lack of a significant difference in BSI scores between the two conditions might be due to the fact that the BSI score decrease is totally or partly caused by a placebo-effect, which is mostly caused by hope and beliefs (Mommaerts and Devroey, 2012). A meta-analysis of all intent-to-treat person-level longitudinal data of major depressive disorder from 16 randomized controlled trials of fluoxetine hydrochloride and 21 adult trials of venlafaxine hydrochloride, resulted in an estimated 78.9% decrease in the probability of suicidal risk for control patients after 8 weeks of study participation and a 90.5% decrease for treated patients (Gibbons et al., 2012). Of note, Iovieno and Papakostas (2012) described that higher placebo response rates are correlated with a lower probability to detect a statistically significant superiority of the drug vs. placebo. Since our study and the before-mentioned meta-analysis from Gibbons et al. (2012) also found a high placebo (sham) response rate on BSI scores, this might explain the absence of significant differences between the active and sham group after 1 week. Between T3 and T4 there was no more intervention and no contact between the patients and the caregivers. Interestingly during those 2 weeks a further significant decrease of BSI scores was obtained. This suggests that the contact with caregivers is not the major cause of the improvement.

To further investigate the effects of this accelerated intermittent Theta Burst protocol, we analyzed the results 1 month after baseline (T4) and found that 39% of these therapy-resistant depressed patients responded to the stimulation protocol in terms of their depressive symptoms which is on its own a very interesting finding. The decrease in suicide risk was not statistically different between the group of HDRS-responder vs. the HDRS-non-responder group, indicating that those patients who showed less than 50% decrease in HDRS-score, defined as “HDRS-non-responders”, did however show a significant decrease in BSI score. Moreover, the improvement in suicide ideation lasted up to 1 month after baseline.

The use of a randomized, sham-controlled design in therapy-resistant and antidepressant-free patients contributes to the methodological strengths of this study. In addition, the left DLPFC stimulation site was targeted using a neuronavigation system which is more precise than the standard 5 cm technique that has been applied in all mentioned previous studies (Fitzgerald et al., 2009; Holtzheimer et al., 2010 ; Hadley et al., 2011; Keshtkar et al., 2011; Wall et al., 2011; George et al., 2014). Moreover, our sample size is relatively large, especially taking into account the difficulties in recruiting therapy-resistant depressed and suicidal patients that are antidepressant-free, such as ethical and safety issues, and also compared to other studies (Hadley et al., 2011; Wall et al., 2011). On the other hand it is rather small from a statistical point of view.

Another strength is the fact that we selected a diagnostically homogeneous group of unipolar depressed patients with suicidal ideations where other studies of interventions for suicide ideations often combine unipolar and bipolar depressed subjects or include non-depressed personality disordered suicidal patients or patients with other comorbidities (Hadley et al., 2011; Wall et al., 2011; George et al., 2014). We used an extensive suicide assessment scale to assess the suicide risk instead of only one suicide item of a depression rating scale, as has been done by other research groups (O’Reardon et al., 2007; Holtzheimer et al., 2010; Keshtkar et al., 2011). The evaluation at 1 month after baseline and 6 months after baseline is longer than what was reported in most previous studies. Furthermore, iTBS was administered in monotherapy, i.e., without concomitant anti-depressant medication whereas other studies applied rTMS as an adjunctive treatment (Holtzheimer et al., 2010; Hadley et al., 2011; Keshtkar et al., 2011; Wall et al., 2011; George et al., 2014). This made it possible to evaluate the accelerated iTBS protocol without the confounding factor of another treatment. Taking into account the potentially large placebo-effects in this population, the treatment protocol has the advantage over previous reports in being sham-controlled (Brunoni et al., 2009; Gibbons et al., 2012).

A possible pitfall is the cross-over design which necessitates the evaluation of the difference between active and sham stimulation on T2 in order to avoid carry-over effects that could be expected during the second week. As mentioned, the maximal effect of the treatment is not expected so quickly (i.e., T2 is only 3 days after the last stimulation session) so this evaluation possibly comes too soon to find a significant difference between these two groups. This assumption is based on the proposed neurophysiological effects of TBS: it is thought to create more robust neuroplasticity effects, which possibly become apparent later in time after treatment. (Chung et al., 2015) Interestingly, between T3 and T4 there was no more stimulation and no contact between the patients and the caregivers. And during those 2 weeks a further significant decrease of BSI scores was obtained. On the other hand, it would have been unethical to choose not to treat one group of these severely ill patients to obviate the cross-over protocol or to wait any longer between the two conditions. Although the BSI is a valid and commonly used instrument, scoring assumes that the total score equals to zero if the patient responds “no” to the first five screening questions. Consequently it quickly turns to a score of “zero” and the range of low scores is limited, therefore possibly limiting the interpretation possibilities.

A disadvantage of the therapy-resistance and lack of concomitant antidepressant, antipsychotic or mood-stabilizing treatment is the fact that our patient sample is less generalizable to a clinical population of suicidal patients that arrive at the emergency ward.

The findings from this study show that this accelerated iTBS protocol is safe to be applied to depressed, suicidal patients and that there is an important effect of sham stimulation. The iTBS protocol also resulted in a significant decrease of suicide risk in depression-non-responders. Further research is necessary to confirm these findings. Such research should be performed in sham-controlled designs in order to study the effects of rTMS in monotherapy and, when confirmed, in more naturalistic conditions.

## Conclusion

Suicide is a major health concern and effective interventions to rapidly reduce suicide risk are lacking. The currently used accelerated iTBS protocol appears to be a feasible and safe treatment for depressed patients with suicidal ideation. The protocol was associated with a significant decrease in suicide risk, lasting up to 1 month, unrelated to active or sham stimulation and unrelated to depression-response. One should thus note an important effect of sham stimulation. Interestingly, the observed decrease in suicide risk was not only due to an improvement of depression and 2 weeks after treatment a persistent decrease of suicidal thoughts was found even in depression non-responders. The current study has the advantage above previous studies in using a sham-controlled design to study the effects of neuronavigated accelerated iTBS in an antidepressant-free diagnostically homogenous group. rTMS should be further investigated as an acute intervention for suicide risk in unipolar depression in sham-controlled trials.

## Author Contributions

All authors: substantial contributions to the conception or design of the work; or the acquisition, analysis, or interpretation of data for the work; and drafting the work or revising it critically for important intellectual content; and final approval of the version to be published; and agreement to be accountable for all aspects of the work in ensuring that questions related to the accuracy or integrity of any part of the work are appropriately investigated and resolved.

## Conflict of Interest Statement

The authors declare that the research was conducted in the absence of any commercial or financial relationships that could be construed as a potential conflict of interest.
